# Preventing at-risk children from developing antisocial and criminal behaviour: a longitudinal study examining the role of parenting, community and societal factors in middle childhood

**DOI:** 10.1186/s40359-018-0254-z

**Published:** 2018-08-10

**Authors:** Madeleine Stevens

**Affiliations:** Personal Social Services Research Unit, London School of Economics and Political Science, Houghton Street, London, WC2A 2AE UK

**Keywords:** Parenting, Conduct disorders, Behaviour problems, Family support, Social work, ALSPAC, Antisocial behaviour, Prevention, Social support

## Abstract

**Background:**

Many childhood risk factors are known to be associated with children’s future antisocial and criminal behaviour, including children’s conduct disorders and family difficulties such as parental substance abuse. Some families are involved with many different services but little is known about what middle childhood factors moderate the risk of poor outcomes. This paper reports the quantitative component of a mixed methods study investigating what factors can be addressed to help families improve children’s outcomes in the longer term. The paper examines six hypotheses, which emerged from a qualitative longitudinal study of the service experiences of eleven vulnerable families followed over five years. The hypotheses concern factors which could be targeted by interventions, services and policy to help reduce children’s behaviour problems in the longer term.

**Methods:**

The hypotheses are investigated using a sample of over one thousand children from the Avon Longitudinal Study of Parents and Children (ALSPAC). Multiple logistic regression examines associations between potentially-moderating factors (at ages 5–10) and antisocial and criminal behaviour (at ages 16–21) for children with behaviour problems at baseline.

**Results:**

ALSPAC analyses support several hypotheses, suggesting that the likelihood of future antisocial and criminal behaviour is reduced in the presence of the following factors: reduction in maternal hostility towards the child (between ages 4 and 8), reduction in maternal depression (between the postnatal period and when children are age 10), mothers’ positive view of their neighbourhood (age 5) and lack of difficulty paying the rent (age 7). The evidence was less clear regarding the role of social support (age 6) and mothers’ employment choices (age 7).

**Conclusion:**

The findings suggest, in conjunction with findings from the separate qualitative analysis, that improved environments around the child and family during middle childhood could have long-term benefits in reducing antisocial and criminal behaviour.

## Background

Primary-school-age children with symptoms of conduct disorders are at high risk of later antisocial and criminal behaviour [1, 2]. However the causal pathways are varied and complex and many children are resilient to the presence of risk factors and do not experience negative outcomes [3]. Whether or not children go on to display such behaviour is associated with a wide range of childhood factors including social and emotional characteristics of the child [4], community, neighbourhood [5] and school factors [6].

Much research has focussed on the role of parenting behaviours and a meta-analysis of 161 papers found the parenting factors most strongly linked to children’s later delinquency were parental monitoring, psychological control, rejection and hostility [7]. However the majority of included studies were cross-sectional and it is possible that other factors are the cause of both the parenting behaviours and children’s conduct problems, including environmental factors, such as social and economic pressures, as predicted by family stress models [8]. Shared genetic factors may also play a role [9], and genetic influences on behaviour can contribute to explanations of the apparent heritability of environmental stressors linked to conduct problems, such as maternal negativity and negative life events [10].

By primary-school age many of the risk factors for antisocial behaviour, including conduct problems, are apparent, but although some families are involved with many services, we know very little about their long-term impact [11]. Quality of parenting is often seen as the most easily modifiable of the influences affecting children’s behaviour as well as a host of other developmental outcomes and life opportunities [12]. Controlled trials have shown short-term improvements in children’s behaviour following parenting programmes in reducing harsh parenting practices and children’s behaviour problems in the short term [13, 14]. However, the most hard-to-help families are missing from research examining effectiveness of interventions and little is known about what aspects of support might be most likely to improve outcomes [15, 16].

Intervention could also target a wider range of determinants of parenting capacity. Research has suggested that a number of factors predict positive parenting practices including social support during pregnancy and mother’s age [17]. Factors associated with poor parenting include mental health problems, poor housing, poverty and unemployment [18]. Quantitative as well as qualitative findings have suggested that informal support may be protective [19, 20].

Much of the evidence of effectiveness for current favoured preventative approaches uses study designs which take little account of contexts, and of the multitude of service and other influences affecting families’ experiences and wellbeing [13, 21]. These influences can include interactions with services and agencies in education, health, social care, criminal justice, housing, parenting, benefits, voluntary/community groups and the private sector (e.g. money-lenders and landlords) as well as relationships within the family and in the wider community, and potential causal factors such as health, emotional/psychological and environmental characteristics and lack of resources and skills [22].

### The current study, and the qualitative study which informs it

This study aimed to contribute to the evidence base by looking at what factors, which can be targeted by interventions, services or policy, affect children’s antisocial and criminal behaviour in the longer term. The analysis is informed by an in-depth qualitative longitudinal study of the experiences of a small group of families in difficulties followed over five years. The qualitative study aimed to investigate how families with children at risk of future antisocial and criminal behaviour benefit, or fail to benefit, from the various types of intervention they come into contact with. Qualitative analysis of the families’ accounts, and the accounts of practitioners families nominated as helpful, is presented elsewhere, and suggested factors influencing family functioning and child behaviour over the five years [23].

In some cases, the factors relate to *change*s occurring during the school years. For example, the likelihood of children being involved in antisocial or criminal behaviour in the future may be reduced if parents become less hostile towards their child, or give attention to their own mental health and therefore become better able to deal with their child’s behaviour.

The qualitative analysis also highlighted the possible risks and benefits, for children’s behaviour and family functioning, of neighbourhood factors, and of mothers’ social network, housing, work and money issues. A number of the mothers in the qualitative study praised the tolerance of their neighbours. The analysis suggested that if mothers felt their neighbourhoods were good places to live, it could benefit family wellbeing and child behaviour, and that, conversely, lack of social support could be a risk factor. However, aspects of social networks could also have negative impacts, for example, other families experiencing difficulties could create further burdens on study mothers, and some friendships could exacerbate negative attitudes and behaviours towards services and practitioners, as well as sometimes exposing study family members to inappropriate behaviours. Many mothers said they would like to work but that it was not possible because of the demands of looking after the child, and money worries, particularly where housing was affected, were a source of maternal stress.

The current study explores whether these school-age factors are related to children’s development of antisocial behaviour in the longer term. Themes from the qualitative analysis are investigated quantitatively using a rich, longitudinal set of data from a larger group of families with children with difficult behaviour. The analyses test hypotheses for those themes which could be approximated with data from the Avon Longitudinal Study of Parents and Children (ALSPAC).

## Methods

The aim of the analyses was to investigate the following hypotheses. The hypotheses all relate to aspects of the environment around children with behaviour problems between ages 5 and 11. The later outcomes referred to in relation to antisocial behaviour are measured between the ages of 16 and 21:

- Hypothesis 1: Children whose mothers become less hostile towards them are less likely (compared to those whose mothers remain hostile) to display antisocial behaviour in the future.
- Hypothesis 2: Improved maternal mental health during the primary school years reduces the chance of children going on to display antisocial behaviour.
- Hypothesis 3: Children whose mothers consider their neighbourhood a good place to live are less likely than others to display antisocial behaviour in the future.
- Hypothesis 4: Children whose mothers have more social support are less likely to display future antisocial behaviour.
- Hypothesis 5: Children whose mothers are not working by choice, compared to those with mothers who would prefer to be in employment, are less likely to display later antisocial behaviour.
- Hypothesis 6: Children of mothers who have no difficulty paying rent when the child is primary school age are less likely than others to go on to have antisocial behaviour.

### Data

The analyses made use of data from the prospective UK birth cohort, the Avon Longitudinal Study of Parents and Children (ALSPAC) which follows mothers and their children who were born in 1991–1992, from pregnancy up to the present day (for more details see http://www.bristol.ac.uk/alspac). The ALSPAC website contains details of all the data that is available through a fully searchable data dictionary (www.bris.ac.uk/alspac/researchers/data-access/data-dictionary/). The analyses use a subsample of ALSPAC children with problem behaviour in primary school (ages 5–11) as described below. This subsample was used to allow examination of potentially protective factors specifically for children with primary school-age behaviour problems, rather than for children in general.

#### Behaviour problems, ages 5 to 11

Cases were considered to have primary-school-age behaviour problems, and were therefore included in the analysis, if the ALSPAC child met any of the following criteria.

- Scores of 4 or above, indicating presence of conduct problems, on at least one of the Strengths and Difficulties Questionnaire measures (conduct problems sub-scale) [24] completed by mothers at average child ages of 6.7, 8 and 8.7 years and by teachers of children in school years three (age 7–8) and six (age 10–11).
- Meets clinical definition of oppositional or conduct disorder according to the Development and Well-Being Assessment (DAWBA) which uses combined clinic assessment and parent and teacher reports at age 7 [25].
- Identified as having disciplinary problems at school, according to parent-report at age 9.
- Child expelled from school, by age 8.5, according to parent report.

Children with conduct problems identified at any of these primary-school timepoints were included to address the problems of missing data in ALSPAC. ALSPAC participants are asked to complete questionnaires at many timepoints, and parents of children with conduct problems, as well as parents with a range of socio-demographic disadvantages, are more likely to have missed some questionnaires, as well as being more likely to drop out of the study completely [26].

#### Outcome measure: antisocial and criminal behaviour (ASB)

A single summary binary variable was constructed to indicate whether the young people had displayed antisocial behaviour at any of the five timepoints between ages 16 and 21 at which the relevant questions were asked. When ALSPAC children were 16 parents reported on their child’s behaviour, while the other four question sets were answered by the young people themselves, usually by postal questionnaire, but, at age 17, by computer during a clinic session. In four question sets, including the parent-reported set, respondents were asked about the number of times they (or their child) had been involved in a variety of antisocial or criminal behaviours in the past year, e.g. stolen something from a shop, threatened to hurt someone, actually hurt someone, deliberately damaged property. The scale is based on the *volume of offending* measure used in the Edinburgh Study of Youth Transitions and Crime [27]. A case was considered to have an outcome of antisocial and criminal behaviour (ASB) if they scored in the top 10% of the full ALSPAC sample on any of the four scales. In the fifth question set (age 17) respondents were asked about involvement with the criminal justice system, and were considered to have an ASB outcome if they had been charged for a crime or given an official caution or fixed penalty notice by police, a Court fine or Antisocial Behaviour Order, or had spent time in a Secure Unit, Young Offenders Institution or prison. Fifteen per cent of the full ALSPAC sample meet the criteria for antisocial behaviour, a cut-off level used elsewhere [28].

#### Predictor variables

A set of predictor variables was identified (defined below), representing the modifying factors suggested by the qualitative analysis and reflected in the hypotheses listed above.

#### Reduction in maternal hostility

In ALSPAC, parents were asked about their attitudes towards their children at ages 4 and 8. Responses to the following items have been used previously, supported by factor analysis results, to measure parental hostility [29]:

*I often get very irritated with this child*

*I have frequent battles of will with this child*

*This child gets on my nerves*

Responses could be coded 2 (yes), 1 (sometimes) or 0 (no) and were summed to make a scale of 0–6. Scores of 5 or 6 represent high maternal hostility towards the child. Mothers were defined as having become less hostile if their hostility reduced from high to lower levels.

#### Improved maternal mental health

Mothers’ depression was measured postnatally and when children were aged six and ten using a ten-item scale constructed from a validated psychometric questionnaire, the Edinburgh Postnatal Depression Scale (EPDS) [30] and used as a continuous variable. The hypothesis concerned the effect of *change* in mother’s depression on children’s later antisocial behaviour. Change in mother’s depression, the difference between scores on the EPDS at two ages, is the predictor, and mothers’ baseline level of depression is controlled for through inclusion as a covariate.

#### Neighbourhood is a good place to live

Parents were asked their opinion of their neighbourhood as a place to live when children were aged 5, 7 and 10. A binary variable indicates whether the opinion was Good (combining responses ‘good’ and ‘fairly good’) or Not good (combining responses ‘not very good’ or ‘not good at all’).

#### Social support

Questions about parents’ social support and social network were asked when children were aged 5, 6 and 12. A social support scale, providing a continuous variable for use in analyses, was constructed at each age from responses to a 10-item inventory that assessed whether parents experienced emotional support (e.g. sharing feelings, being understood) and instrumental support (e.g. others helping with tasks, providing financial help if needed) from partners, neighbours, friends and family [17]. A separate continuous measure, for social networks, was similarly derived from responses to items about numbers of friends and family and frequency of contact.

#### Not working by choice

When ALSPAC children are aged 7 their mothers are asked whether they are working, and if not, whether this is by choice.

#### Difficulty paying rent

When ALSPAC children are aged 7 their mothers are asked about the level of difficulty they face in paying their rent. A binary variable is used to indicate difficulty, combining responses ‘slightly’, ‘fairly’ or ‘very’ difficult versus those who answered it was ‘not difficult’.

#### Covariates

Potential confounders of the relationship between hypothesised predictors and ASB were included where data considerations allow. For all analyses it was important to adjust for the level of children’s behaviour problems at primary school. Age six behaviour problems was chosen as this was the first measure taken after starting primary school. All other confounders for potential inclusion in analyses were measured before the age of starting school.

Variables were chosen as covariates if they were likely to be alternative predictors of the outcome which may be confounded with the hypothesised protective factor, based on previous research (e.g. [31, 32, 35]) and examination of associations in the current sample.

The stressful life events score is based on responses to an inventory of potentially stressful events when the child is age 47 months. Mothers indicate whether the event occurred and the degree to which it affected them. The score is derived for ALSPAC based on previous inventories [33, 34]. The financial difficulties score is constructed in the ALSPAC dataset, derived from responses, when the child is aged 33 months, to a series of questions about degree of difficulty affording various essential items; higher scores indicate more difficulty. Tables 1 and 2 compare these characteristics for those young people who do or do not display later ASB. The tables show that young people in the ASB group are more disadvantaged on every relevant variable.

**Table 1:** Comparison of key covariates (categorical variables) for children with behaviour problems ages 6–10, comparing those who go on to have antisocial behaviour (ASB) with those who do not

Categorical variables	Child’s age at measurement	Categories	No ASB age 16–21		ASB age 16–21		Chi-square(df) and *p* values^a^
			n	(%)	n	(%)	
Child’s sex	Birth	Male	472	(51.8)	193	(57.1)	χ^2^(1) = 2.77 *p* = 0.096
		Female	439	(48.2)	145	(42.9)	
Biological father lives with child	47 months	No	95	(11.8)	54	(17.9)	χ^2^ = (1)7.08 *p* = 0.008
		Yes	710	(88.2)	247	(82.1)	
Housing owned or not	33 months	Not owned	133	(16.5)	104	(33.4)	χ^2^ = (1)38.26 *p* < .001
		Owned	671	(83.5)	207	(66.6)	

^a^comparing ASB groups

**Table 2:** Comparison of key pre-baseline and conduct problems covariates (scale variables) for children with behaviour problems ages 6–10, comparing those who go on to have antisocial behaviour (ASB) with those who do not

Scale variables	Child’s age at measurement	No ASB age 16–21	ASB age 16–21	t(df)	p^a^	95% CI of difference	N
		Mean (sd)	Mean (sd)				
Mother’s age	Birth	29.0 (4.6)	28.2 (4.9)	2.79(1207)	0.005	0.25,1.43	1209
Stressful life events score	47 months	13.6 (10.7)	17.1 (12.0)	−4.72(1117)	< 0.001	−4.96,-2.05	1119
Financial difficulties	33 months	3.3 (3.7)	4.6 (4.3)	−5.24(1108)	< 0.001	−1.91,-0.82	1110
Conduct problems	6 years	3.26 (1.62)	3.66 (1.58)	−3.68(1108)	< 0.001	−.062,-0.19	1090

^a^Unpaired t tests

Covariates of theoretical relevance to each hypothesis were included in two ways. Firstly, each covariate was entered individually along with the predictor (if preliminary analyses had shown a statistically significant association between the two). Secondly, all covariates which had retained a significant association with ASB when included individually with the predictor were entered together. The aim was to achieve a parsimonious set of models retaining statistical power and transparency of interpretation.

### Analysis

Relationships between predictor variables and antisocial behaviour were first examined visually and then compared with simple two-variable analyses, prior to running multivariate logistic regressions to control for potential confounders. For the binary predictor variables differences between cases with and without ASB at ages 16–21 were examined in cross-tabulations and assessed using chi-square tests. For scale predictor variables distributions were compared using means and standard deviations, and differences in means were tested using unpaired t tests.

Regressions were carried out, using Stata 14 [35], both unadjusted, and adjusted for covariates which could confound any association between the hypothesised predictors and ASB. Potential covariates were chosen based on existing knowledge about factors associated with antisocial behaviour (see for example [36]) in order to control, as far as possible, for confounding background factors and focus on the impact of school-age factors. Sex is recorded in ALSPAC at birth, and so is the variable adjusted for rather than gender. For the adjusted analyses only those children with a conduct disorder measure at age 6 were included, so that age 6 conduct problems could be adjusted for. A *p* value below 0.05 is referred to as indicating a statistically significant association, although it is acknowledged that this is an arbitrary cut-off [37]. To retain cases in the analysis, scores were estimated, if fewer than half the responses were missing, using existing items and adjusting for the number of items (prorating).

## Results

The sample consisted of 1249 children (53% male) with behaviour problems at primary school age and who had data available on their antisocial behaviour between the ages of 16 and 21. This constitutes 17% of the 7253 ALSPAC children with a measure of primary-school age behaviour problems and a measure of adolescent antisocial behaviour as defined above. Twenty-seven per cent of this behaviour problems sample display antisocial behaviour at ages 16–21 (*n* = 338). This compares to 13% of ALSPAC children who did not have primary school-age behaviour problems (Chi square(1) = 170.6, *p* < 0.001) and display antisocial behaviour at ages 16–21.

The sample represents only 51% of those with behaviour problems at primary school age, because of the high rates of ALSPAC drop-out and non-response in adolescence. Comparison between those with and without an available ASB measure in adolescence shows that those with available ASB data (the sample for the current study) are more likely to be girls (47% versus 27%; *p* < 0.001), while their mother is likely to be older (mean 28.8, versus mean 26.2, *p* < 0.001) have fewer financial difficulties (mean 3.6, versus mean 4.6, *p* < 0.001) and be a homeowner (79% versus 59%, *p* < 0.001). However there is no difference in the age 6 behaviour scores between those with and without ASB data (included sample mean 3.37, sd 1.62 versus mean 3.42, *p* = 0.52).

Descriptive data comparing hypothesised modifying factors for those with, and without, antisocial behaviour at ages 16–21 are shown in Table 3 (categorical predictor variables) and Table 4 (continuous predictor variables). Table 3 shows the percentage of children with behaviour problems at primary-school age who went on to have ASB at ages 16–21 in each category. Table 4 compares mean values of the continuous predictor variables for those who did or did not have later ASB. Sample sizes are different for each predictor variable because of missing data and because some predictors only concern sub-samples. Only mothers with high maternal hostility when children were aged 4 were included in the reduced maternal hostility analysis (*n* = 297) and only non-working mothers who answered the question about not working by choice were included in that analysis (*n* = 282).

**Table 3:** Categorical predictor variables and antisocial and criminal behaviour (ASB) age 16–21

Categorical predictor variables	n (%) with age 16–21 ASB		Total with predictor	Chi square and *p* values
Change in maternal hostility (age 4 to 8)				
Reduced hostility (between ages 4 and 8)	27	(22)	121	χ^2^(1) = 6.66, *p* = 0.010
Hostility remains high	64	(36)	176	
Opinion of neighbourhood as a place to live, child age 5				
Good	271	(26)	1027	χ^2^(1) = 7.58, *p* = 0.006
Not good	27	(42)	64	
Difficulty affording rent, child age7				
No difficulty	170	(23)	735	χ^2^(1) = 15.23, *p* < 0.001
Difficulty	78	(37)	214	
Non-working mother choice, child age 7				
Chose not to work to stay at home with child	56	(26)	218	χ^2^(1) = 1.9, *p* = 0.172
Did not choose not to work	22	(34)	64

**Table 4:** Scale predictor variables and antisocial and criminal behaviour (ASB) age 16–21

Predictor variable	Child age	No ASB			ASB			Unpaired t test		
		Mean score	SD	n	Mean score	SD	n	Mean difference	p	95% CI
Depression (EPDS)	6	5.7	3.9	781	6.5	4.2	296	0.8	0.004	0.26, 1.32
Depression (EPDS)	10	5.3	4.1	787	6.1	4.5	297	0.8	0.007	0.22, 1.35
Social support	6	16.8	4.6	774	16	4.8	294	0.78	0.015	0.15, 1.40
Social network	6	22.2	4.3	777	21.5	4.7	295	0.69	0.023	0.10, 1.28

*EPDS* Edinburgh Postnatal Depression Scale; higher score = more depressive symptoms

The tables show that for every hypothesised predictor those exposed to the hypothesised protective category of the predictor were less likely to have later ASB. The *p* values testing these relationships are all below 0.05 except for mother’s choice of not working. Examination of the variable ‘mother is in paid employment’ in ALSPAC shows no association with the ASB outcome (*p* = 0.591). The difference in the likelihood of antisocial behaviour between children of non-working mothers who did or did not choose to stay at home with the child is not strong (*p* = 0.172, Table 3). Numbers are small, and the difference quite large, but there is insufficient evidence to support Hypothesis 5 and so this predictor was not further investigated in the regression analyses.

The remaining predictor variables were further investigated in logistic regression analyses. Although all ALSPAC children in the analysis met the cut-off for conduct problems at least at one primary school age timepoint, level of baseline (age six) conduct problems differed between those who did or did not display ASB at ages 16–21. Therefore, it was important to examine the strength of associations adjusted for baseline conduct problems. Where the association between the predictor and ASB remained significant further potentially confounding variables were included in the analyses (Table 5) as described above.

**Table 5:** Logistic regressions showing impact of hypothesised predictors in reducing antisocial and criminal behaviour (ASB)

Predictor	Unadjusted/Adjusted for:	Odds Ratio	p	95% CI	N
Reduced maternal hostility age 8 (subsample with hostile mothers at age 4)	Unadjusted	0.50	0.010	0.30, 0.85	297
	Conduct problems age 6	0.57	0.042	0.33, 0.98	287
	Entered together: Conduct problems age 6 Financial difficulties Housing tenure Biological father lives with child age 4 Mother’s age Stressful live events	0.45	0.008	0.24, 0.81	276
Change in depression score (age 6 – age 10)	Depression age 6	0.98	0.213	0.94, 1.01	979
Change in depression score (postnatal – age 10)	Postnatal depression	0.95	0.012	0.92, 0.99	1034
Change in depression score (postnatal – age 10)	PND and conduct problems age 6	0.95	0.009	0.92, 0.99	949
Change in depression score (postnatal – age 10)	Entered together:	0.95	0.009	0.91, 0.99	885
	Postnatal depression				
	Conduct problems age 6				
	Child’s sex				
	Housing tenure				
	Financial difficulties				
	Stressful life events				
Neighbourhood is a good place to live, age 5	Unadjusted	0.49	0.007	0.29, 0.82	1091
Neighbourhood is a good place to live, age 5	Conduct problems age 6	0.57	0.047	0.32, 0.99	1030
Social Support age 6	Unadjusted	0.96	0.015	0.94, 0.99	1068
	Conduct problems age 6	0.98	0.149	0.95, 1.01	1024
Social Network age 6	Unadjusted	0.97	0.023	0.94, 1.00	1072
	Conduct problems age 6	0.98	0.180	0.95, 1.01	1027
Can afford rent	Unadjusted	0.52	0.000	0.38, 0.73	949
	Conduct problems age 6	0.54	0.000	0.38, 0.75	917
	Entered together:				
	Conduct problems age 6	0.65	0.021	0.46, 0.94	863
	Housing tenure				
	Stressful life events				
	Mother’s age				

Table 5 confirms the statistically significant relationship between each of these predictor variables and ASB before adjustment for potentially confounding factors, and shows the effect on the odds ratio after adjusting for children’s level of behaviour problems at age six. All the adjusted odds ratios indicate that children exposed to the protective factor are less likely to display later antisocial and criminal behaviour. However, for some of the hypothesised predictors, the 95% confidence interval of the odds ratio indicates a non-statistically significant association. Nevertheless, reduction in hostile parenting (Hypothesis 1), lower rates of maternal depression compared to postpartum (Hypothesis 2), good feelings about the neighbourhood (Hypothesis 3), and ease of paying the rent (Hypothesis 6) are all associated with a lower likelihood of antisocial behaviour (with *p* values lower than 0.05) after adjusting for the level of baseline behaviour problems.

The associations between antisocial behaviour and less hostile parenting (change between when child was aged four and aged eight), improved parental mental health (compared to the postnatal period, but not between when children are aged six and aged ten) and difficulty paying the rent, remain statistically robust when the role of additional background covariates is taken into account.

Regarding maternal depression, it is possible that change over four years (between ages six and ten) is not long enough to see any effect on children’s later antisocial behaviour outcomes. Analysis of a sub-group of 185 mothers with high depression at child’s age six, confirmed this result: children of mothers whose depression improved between when their child was age six and age ten are no less likely to have later antisocial behaviour than those whose mothers remain depressed at age ten (*p* = 0.63).

A subsequent analysis looked at change in mother’s depression score between eight weeks postpartum and child’s age ten, controlling for baseline (postpartum) depression score. This change, over ten years, is significantly related to children’s later antisocial behaviour (Table 5) with a reduction in mother’s depressive symptoms being associated with a lower likelihood of the child developing antisocial behaviour, even after controlling for relevant background factors.

Children of mothers who felt their neighbourhood was a good place to live were less likely to display later antisocial behaviour, even after adjustment for children’s level of behaviour problems. However, the association is reduced when adjusting for earlier stressful life events and is no longer statistically significant after adjusting for housing tenure at birth.

Adjusting for children’s level of conduct problems at age six, there was insufficient evidence to conclude that their later ASB is predicted by mothers’ social support or social network (Hypothesis 4). *Changes* in social support were also examined but no statistically significant associations with ASB were found. Adjusting for any covariate other than child’s sex reduced the statistical significance of the associations indicating that these other family characteristics are stronger predictors of later ASB than social support and social network.

Ease of affording rent remains a highly significant predictor of ASB status when adjusting for a number of family background variables including mother’s mental health at child’s age six; as shown previously, mother’s depression alone is a statistically significant predictor of ASB (OR = 1.05, *p* = .004). Mother’s depression becomes a less significant predictor when entered in logistic regression with ‘ease of affording rent’ (OR = 1.03, *p* = .095). Mother’s depression at child’s age six is also a strong predictor of ease of paying rent at age 7 (OR = .89, *p* < 0.001) suggesting that financial stresses such as difficulty paying rent may partially mediate the relationship between mother’s depression and ASB. Difficulty paying the rent remains a significant predictor of ASB after adjusting for behaviour problems, mother’s age and early childhood housing tenure and stressful life events.

## Discussion

The underlying interest of this study is in how families and children can be helped and supported, during the school years, to prevent at-risk children developing antisocial behaviour. Therefore, although there is evidence that many factors (including the covariates presented above) are associated with children’s later antisocial behaviour, of particular interest is any evidence that *change* in the hypothesised factors, during the school years, is linked to lower risk of antisocial behaviour.

The finding that mothers’ reduced hostility towards their child appeared to have lasting associations with children’s later antisocial and criminal behaviour supports existing findings of cross-sectional associations between parenting behaviours and child outcomes [38]. The longitudinal finding has important implications for preventative efforts, suggesting that intervention to support relationships between parents and their children could have long-term effects. The qualitative analysis which informed the current study [23] suggested that reduced hostility could be brought about when mothers gained empathy for their child through therapeutic intervention, vastly improving family relationships.

However, helping mothers to feel less hostility towards their child is complex. The qualitative study showed that intervention that, either implicitly or explicitly, blames mothers for children’s behaviours can be counter-productive if parents are not empowered to make changes. Parenting behaviours of stressed and distressed mothers can easily divert from practitioners’ view of good parenting [39] and professionals’ behaviours can increase, as well as reduce, resistance to change [40]. Common stages in processes of behaviour change have been found to apply to mothers facing child protection intervention: resistance, ambivalence, motivation, engagement and action [18]. Intervention which helps mothers improve their mental health and ‘readiness to change’ may be a first step before parenting issues can be tackled [41].

The high prevalence of mental health problems among parents of children referred to mental health services is known [42], as is its relationship with parenting [43], and with children’s outcomes [44]. The ALSPAC analysis showed not only that mothers’ mental health during primary school was related to children’s later antisocial behaviour, but also that improvements in maternal mental health (compared to postpartum) may be protective. The role of changes in maternal mental health occurring over a four-year period *during* the primary school years was less obvious however.

Many factors have been found elsewhere to be predictive of mothers’ depression including those examined here: neighbourhood, mothers’ social support, voluntarily unemployed status, and ease of paying the rent. Neighbourhood danger appears to exacerbate negative impacts of harsh parenting on conduct disorders in children [45] but neighbourhood cohesion can moderate harsh parenting’s effects [46]. Although a statistically significant association was not found in the present analysis, much research has pointed to the protective role of supportive social networks [47, 48]. The qualitative analysis showed the important, but complicated, role of social networks in helping a family in difficulties to bring up a difficult child. Wider family and social connections could be a crucial support but in some cases could be more of a hindrance. Relationships between potentially protective factors and outcomes can be difficult to tease out in survey data.

Similarly, it is possible, as indicated in the qualitative analysis of interviews [23], that there could be both positive and negative effects of mothers’ work on child behaviour, which was not shown to be related to children’s future antisocial behaviour in the ALSPAC study. In the qualitative study sample of eleven families only one mother was working by the final follow-up, and several had had to give up work, or said they could not enter paid employment because of the demands of their child, for example being frequently requested to collect them early from school, or to keep them at home when excluded. Parents regretted this as they felt paid work would improve their own wellbeing and be a good example to their children, but two parents suggested that they would be worse off financially and subject to additional stressors if they entered paid work.

The findings reported here suggest a variety of different factors which could be targeted by intervention to improve outcomes for children and help prevent antisocial behaviour. Research on family resilience has pointed to the danger of a ‘narrow focus on parental pathology’ obscuring the role of other resources which can be strengthened to improve family resilience [49]. It has been suggested that a focus on the relatively well-evaluated parenting programmes may have restricted availability of alternative forms of family support [50] which are harder to define [51] and evaluate [52]. Evaluations of preventative intervention in the UK have had disappointing results on quantitative comparative results, including evaluations of the Troubled Families Programme, Family Nurse Partnership and Homestart, despite those involved in delivering and receiving the programmes describing the benefits they felt had been achieved [53–57]. Possible explanations include that there really was no positive effect, that the wrong outcomes were measured, that more time was needed for positive outcomes to emerge or that comparison groups were not well matched. Unfortunately these evaluative efforts often have little to say about what aspects of support were helpful for those who *did* benefit.

The present paper suggests the value of a different approach where quantitative analysis is rooted in a qualitative in-depth study of parents’ and practitioners’ experiences, aiming to unearth what was actually helpful for families and then to examine quantitative outcomes in a larger sample with a longer follow-up. The qualitative analysis suggested factors which appear helpful, but other factors which hold back change, uncovering some of the subtleties around need for, and provision of, help which could not be identified in survey data. Although the results of the ALSPAC analysis were mixed there were positive outcomes for some of the factors hypothesised as helpful, suggesting that, with a long enough follow-up, there may be some lasting preventative effect of primary school-age changes in family functioning (reduction in hostility) and factors affecting that family functioning, such as improved maternal mental health and ease of affording the rent.

### Limitations

Despite the richness of the ALSPAC data, only a subset of the themes arising from the qualitative analysis could be investigated. The qualitative and ALSPAC study samples are not perfectly matched, as the qualitative study families all face risk factors additional to the child’s behaviour problems. The most disadvantaged families are underrepresented in ALSPAC [26] and the ALSPAC sample would have become too small for statistical analyses if the same criteria were used. However, ALSPAC family-level risk factors were included as covariates where data allowed. It is possible that the factors which were only weakly supported in the ALSPAC analysis may be more important in a higher need sample. In addition, families in the qualitative study come from two inner and one outer London boroughs, while the ALSPAC families are from the Avon area around Bristol, more diverse in terms of urban or rural location, but less ethnically diverse. Children who were lost to ALSPAC follow-up were more likely to suffer from behaviour disorders than those who did not [58]. Wolke and colleagues found, however, that regression models of predictors of antisocial behaviour were only marginally affected by the non-random nature of attrition [59]. In order to maximise the available sample multiple measures of both behaviour problems and antisocial behaviour were used so that a child needed to have data available on only one of each to be included in the analysis.

The interest of the study is in causality; whether presence of, or improvement in, potentially protective factors during the school years led to improved behaviour in offspring. However, because ALSPAC participants were not randomised, or even assigned, to exposure to the school-age factors of interest it is impossible to say whether the associations observed are due to a causal relationship or whether both result from a third factor. Reverse causality is also possible; despite the temporal ordering employed in the analyses, improvements in children’s behaviour may have led to reduced maternal hostility, or depression. For these reasons, the ALSPAC analysis is rooted in the in-depth qualitative analysis of families’ experiences over five years. While randomised controlled trials provide a way to account for unmeasured differences between groups which may explain different outcomes, they face other constraints which can limit their usefulness for understanding processes of cause and effect in complex, multifactorial real world situations [60]. Despite the limitations of this study’s approach to looking at possible effects of modifiable childhood factors, it would also be problematic to rely only on evidence from trials; this could lead to prioritising interventions which are easier to research, but may not be the most helpful in the longer term. The mixed methods study of which this quantitative analysis was a part, was designed to provide an examination, both in-depth and broad, of what families find useful in bringing about lasting change.

## Conclusions

The ALSPAC analyses presented here show that children who later displayed antisocial behaviour were, on average, disadvantaged on every one of the hypothesised protective factors in middle childhood. These factors can be targeted by intervention, aiming, for example, to improve parent-child relationships, neighbourhood conditions and quality of social support as well as appropriate school-based provision which has not been addressed in this paper ([but see forthcoming paper [61]). The qualitative study on which the ALSPAC analyses were based explored families’ experiences of what helped and what held back improvements in child behaviour and family functioning. Only a subset of the themes from the qualitative analysis could be approximated in the survey data. The qualitative findings help illuminate the meaning of outcomes, such as the lack of quantitative evidence for the impact of improved social support and social network and the possible negative as well as positive outcomes these can bring. The study provides an example of using mixed methods to unpick complex responses to service use while still providing evidence of long-term outcomes.

## Abbreviations

ALSPAC: Avon Longitudinal Study of Parents and Children
HFP: Helping Families Programme
